# An Integrative Approach to Clinical Decision-Making for Treating Patients With Binge-Eating Disorder

**DOI:** 10.3389/fpsyg.2019.02573

**Published:** 2019-11-21

**Authors:** Livia Chyurlia, Giorgio A. Tasca, Hany Bissada

**Affiliations:** ^1^The Ottawa Hospital Research Institute, Ottawa, ON, Canada; ^2^School of Psychology, University of Ottawa, Ottawa, ON, Canada; ^3^Department of Psychiatry, University of Ottawa, Ottawa, ON, Canada

**Keywords:** binge-eating disorder, eating disorders, assessment, patient factors, case formulation

## Abstract

Transtheoretical integrative decision-making models help clinicians to use patient factors that are known to predict outcomes in order to inform individualized treatment. Patient factors with a strong evidence base include: functional impairment, social support and interpersonal functioning, complexity and comorbidity, coping style, level of resistance, and level of subjective distress. Among those with binge-eating disorder (BED), patient factors have not been extensively characterized relative to norms or other clinical samples. We used an integrative decision-making model of these six patient factor domains related to patient outcomes to characterize a sample of 424 adults seeking treatment for BED. Data were from medical charts, a demographics questionnaire, and validated psychometric scales. We then compared these data to published data from normative and other eating disorder (ED) samples. Results showed that the average patient with BED: (1) was significantly more functionally impaired compared to non-clinical norms but somewhat less impaired than other patients with ED, (2) demonstrated clinically significant problems in social support and interpersonal functioning, (3) presented with complex comorbid pathology and high levels of chronicity, (4) used a more internalizing coping style compared to the norm and other ED samples, (5) had low levels of resistance to interventions, and (6) experienced a moderately high level of subjective distress indicating good motivation for treatment. Corresponding recommendations to these findings are that the average patient with BED should be provided higher intensity treatment that is longer in duration, interpersonally focused, directive in nature, and emphasizing self-reflection and insight. Despite the nomothetic nature of the findings, clinicians are encouraged to assess these patient domains when developing an ideographic case conceptualization and to tailor precision treatment to the individual patient with BED.

## Introduction

Binge-eating disorder (BED) is a common eating disorder (ED) characterized by recurrent episodes of binge eating (i.e., eating a large amount of food with a subjective sense of loss of control during the episode) (American Psychiatric Association, 2013). Diagnostic criteria for BED requires that binge-eating episodes occur at least once a week for 3 months, with no inappropriate compensatory behaviors, and that there be marked distress associated with the binge eating (American Psychiatric Association, 2013). Lifetime prevalence estimates of BED are 3.6% among women and 2.1% among men, and point prevalence estimates are 1.7% in women and 0.8% in men (Hudson et al., 2012). Several theoretical models speak to the maintenance and comorbidity of BED, such as the transdiagnostic cognitive-behavioral model (Fairburn et al., 2003; Fairburn, 2008) and the interpersonal model (Wilfley et al., 2000, 2005). Recommended treatment options for BED include psychotherapies such as Cognitive Behavioral Therapy, Interpersonal Psychotherapy, and Dialectical Behavioral Therapy, as well as pharmacotherapy such as some selective serotonin reuptake inhibitors and tricyclic antidepressants (American Psychiatric Association, 2006; Yager et al., 2014). Recent research also indicates the potential utility of lisdexamphetamine (McElroy et al., 2015). Psychological treatment outcomes for those with BED indicate moderate success with approximately 50% of patients abstinent of binge eating at post treatment, but follow up data is limited (Grenon et al., 2018).

For the most part, evidence-based treatment options for BED are identified from randomized controlled trials (RCTs). Results of RCTs speak to the average patient with BED, that is, they take a nomothetic approach, which is useful as a general guide to clinicians. However, these findings do not necessarily take into account individual variability in treatment adherence, treatment processes, and treatment outcomes (Wampold and Imel, 2015). That is, the findings of RCTs do not provide idiographic recommendations to develop a case formulation for precision treatment for a specific patient (Silberschatz, 2017). Patient factors, such as those that we review below account for the largest amount of outcome variance in psychotherapy trials (Norcross and Lambert, 2011). Recently, researchers and clinicians have been increasing efforts to tailor assessment and treatments to specific patient qualities associated with outcomes (Harwood et al., 2011; Silberschatz, 2017; Norcross and Wampold, 2019). Using an integrative approach to consider these variables for clinical decision-making (Beutler et al., 2000) may be useful to assist professionals to make specific treatment decisions for those with BED in order to improve outcomes.

The goal of this study is to better characterize treatment-seeking patients with BED within the framework of an evidence-based, transtheoretical, integrative approach to clinical decision-making (Harwood et al., 2011) using cross-sectional comparisons. Based in part on extensive research of patient factors that impact treatment processes and outcomes (e.g., Norcross, 2011), Harwood et al. (2011) described six key patient qualities for consideration in clinical decision-making: (1) functional impairment, (2) social support and interpersonal functioning, (3) problem complexity/chronicity, (4) coping style, (5) social compliance and resistance, and (6) level of subjective distress. The current study examines how patients with BED function relative to non-clinical and other clinical samples with EDs on these six qualities in order to inform transtheoretical treatment recommendations for those with BED.

*Functional impairment* refers to the degree of disruption to functional roles such as work, social, and educational roles, and reduction in self-care and social responsibilities (Sperry et al., 1996; Beutler et al., 2002). A review of empirical research showed that there is an inverse relationship between level of functional impairment and treatment outcome across various psychological disorders and treatment types (Beutler et al., 2002). Research demonstrates that higher functional impairment can signal the need for medical intervention and/or greater intensity of psychological treatment (Beutler et al., 2002). Both self-report scales and factors such as income, work status, marital status, and level of education can be used to assess functional impairment. While functional impairment is a criterion for several DSM-5 disorders (e.g., major depressive disorder, social anxiety disorder, alcohol use disorder) it is not a specific criterion for BED. Compared to people with obesity, those with BED experience diminished psychosocial functioning, impairments in their ability to carry out everyday tasks, work impairment, and sexual-life impairment (de Zwaan et al., 2003; Rieger et al., 2005; Perez and Warren, 2012). Hay et al. (2017) reported that those with BED had significantly lower income than people without an ED. While functional impairment differences between BED and other ED groups are reported in the literature, very few large-scale studies exist on how those with BED differ from the general population, particularly on employment and marital status, and educational attainment, which are proxies for functional impairment.

*Social support and interpersonal functioning* refers to patients’ current social resources and capacity for social relationships (Harwood et al., 2011). For most mental disorders, low levels of social support are associated with less favorable treatment outcomes and higher rates of relapse (Beutler et al., 2002), and may suggest the need for interpersonally oriented or social-skills-oriented treatment (Harwood et al., 2011). Social isolation, perceived lack of support, interpersonal problems, and insecure attachment style, may indicate that a patient could benefit from improved social skills and interpersonally focused psychotherapy (Castonguay et al., 2006). A study by Ivanova et al. (2017) found that men and women with BED may be less assertive and more socially withdrawn in personal relationships compared to a normative population. Also, insecure attachment is overrepresented in ED populations (Kuipers and Bekker, 2012; Caglar-Nazali et al., 2014). However, there is little published research comparing those with BED to normative non-clinical and other ED samples on measures of attachment insecurity, for example.

*Problem complexity* refers to the number and nature of comorbidities, and can also include chronicity of the presenting problem (Harwood et al., 2011). For most mental disorders, dual diagnoses and longer chronicity predict a poorer prognosis (Beutler et al., 2002), Such patient factors indicate that a longer or more intense course of therapy, or adjunct treatment with medications are required (Beutler et al., 2002). There is extensive reporting of the psychiatric comorbidities found among individuals with BED such as high levels of mood, anxiety, obsessive compulsive, substance-related, bipolar, and personality disorders (Hudson et al., 2007; Grilo et al., 2009; Gerlach et al., 2016). However, those with BED appear to have less comorbidity than individuals with anorexia nervosa (AN) and bulimia nervosa (BN) when it comes to mood, anxiety, and impulse-control disorders according to population-based studies (Hudson et al., 2007) as well as studies using psychometric scales (Tasca et al., 2002). Chronicity of BED is less frequently examined than comorbidities. In one study, earlier age of onset of binge eating predicted worse outcome after treatment (Agras et al., 1995).

*Coping style* refers to typical ways that a patient manages stressors and internal affective experiences (Harwood et al., 2011). Internalizing coping is a tendency to be introverted, withdrawn, and exhibit social restraint, excessive self-attributions, self-blame, and self-criticism (Beutler and Clarkin, 1990). On the other hand, externalizing coping refers to traits of impulsivity, gregariousness, expressiveness, tendency to blame others, tendency to make external attributions of cause, and a need for action (Beutler and Clarkin, 1990). A recent meta-analysis showed a robust relationship between coping style and response to certain therapist stances (Beutler et al., 2018b). Patients with a more internalizing coping style tend to do better with insight-oriented therapy focuses on self-reflection and on interpersonal issues. On the other hand, patients with a more externalizing coping style have better outcomes with therapists who focus on skills-building and symptoms (Beutler et al., 2018b). Very little research exists on the coping styles of those with BED, which would be useful to inform optimal therapist responsiveness.

*Resistance* or social compliance is another patient factor associated with the likelihood of following and accepting a therapist’s treatment recommendations (Harwood et al., 2011). In general, people who are less open to change tend to be less compliant in treatment (Morey, 1991). A recent meta-analysis found that patients higher in resistance received less benefit from psychotherapy, and were more prone to dropping out of treatment (Beutler et al., 2018a). The same review found that patients high in resistance responded better to less directive interventions by psychotherapists, and that patients low in resistance did better in more directive therapies (Beutler et al., 2018a). There is no research that we are aware of that assesses those with BED on their level of resistance or interpersonal compliance.

Level of *subjective distress* is the final patient factor in the integrative approach to clinical decision-making (Harwood et al., 2011), and refers to the subjective ratings of dysphoria or unhappiness felt by the patient. Harwood et al. (2011) view level of distress as an index of motivation and capacity to change. Distress that is too high may be overwhelming and preclude a patient’s ability to make use of treatment, whereas distress that is too low may result in little motivation to engage in therapy (Beutler et al., 2011). Hence there may be an optimum level of distress to indicate good capacity and motivation to change (Harwood et al., 2011). A systematic review by Ágh et al. (2016) reported that patients with BED reported more subjective distress associated with depressive affect and anxiety compared to weight-matched obese individuals. Among those with EDs however, BED is associated with lower risk of suicide and suicidal ideation than AN and BN, suggesting less pronounced subjective suffering (American Psychiatric Association, 2013).

In this study we make use of data from validated measures of personality and psychopathology and other indicators of functioning in order to quantitatively compare a large sample of treatment-seeking individuals with BED to non-clinical and ED groups. We do so to characterize those with BED on the six patient domains that are related to treatment processes and outcomes. These domains were identified by Harwood et al. (2011) and have received empirical support as predictors of patient outcomes (Beutler et al., 2002, 2011, 2018a,b; Beutler and Castonguay, 2006). We hypothesize that: (1) patients with BED will demonstrate higher *functional impairment* compared national and local normative samples; (2) the BED sample will have lower levels of *social support and interpersonal functioning* compared to non-clinical groups, but higher levels compared ED clinical samples; (3) those with BED will have greater *comorbidities and complexity* of concurrent disorders and higher self-reported problems with substance-use compared to the general population, but lower levels compared to ED comparison groups; (4) patients with BED will have higher indices of internalizing *coping style* relative to a normative non-clinical sample (we do not make specific hypotheses about externalizing coping style); (5) the BED sample will score significantly lower than non-clinical populations and other ED groups on measures of *resistance*; and (6) those with BED will score higher on measures of *subjective distress* compared to normative samples, but will score lower than other ED groups.

## Materials and Methods

### Sample

Participants were 424 patients who met Diagnostic and Statistical Manual for Mental Disorders version 5 (DSM-5) criteria for BED (American Psychiatric Association, 2013). All participants were seeking treatment for an ED at a tertiary care center between January 1, 1997 and July 1, 2015. All had an initial consultation appointment, in which they consented to participate in research, and completed a questionnaire package. Sample demographics are presented in Table 1. Comparison groups’ data (i.e., normative samples, other ED groups) included those that were previously published for which we indicate the source of the available data.

**TABLE 1:** Sample demographics and disorder characteristics.

**Variable**	**Total participants (*N* = 424)**
**Mean age ± SD (years)**	39.8 ± 11.4
**Female**	376 (88.7%)
**Race and ethnicity**	
White	303 (71.5%)
Other	30 (7.1%)
Missing	91 (21.4%)
**Disorder status**	
Active	380 (89.6%)
Partially controlled	15 (3.5%)
In remission	29 (6.8%)
**Mean BMI ± SD (kg/m^2^)**	39.9 ± 9.9
**Unemployed**	120 (31.3%)
**Family income (CAD)**	
10,000–29,000	89 (23.6%)
30,000–49,000	72 (19.0%)
50,000–69,000	67 (17.8%)
>$70,000	150 (39.7%)
**Marital status**	
Single	138 (34.8%)
Married	157 (38.9%)
Living together	37 (9.3%)
Divorced	44 (11.1%)
Separated	16 (4.0%)
Widowed	7 (1.8%)
**Highest level of education**	
High school or less	153 (44.5%)
Postsecondary or more	151 (43.9%)
**Psychiatric comorbidity**	
Mood disorder	193 (46.6%)
Anxiety disorder	71 (17.1%)
Substance-related disorder	13 (3.1%)
Adjustment disorder	4 (0.9%)
Psychotic disorder	1 (0.2%)
Other disorder	14 (3.4%)
None	169 (40.8%)

*BMI, body mass index (kg/m^2^); CAD, Canadian Dollars. Sample size for employment status is *N* = 384; Sample size for family income is *N* = 378; Sample size for marital status is *N* = 396; Sample size for highest level of education is *N* = 344; Sample size for psychiatric comorbidity is *N* = 414. The sample used to analyze highest level of education is composed of only patients aged 25–64.*

Inclusion criteria for patients in this chart review was: diagnosed with BED by DSM-IV or DSM-5 criteria, or diagnosed with an eating disorder otherwise not specified (EDNOS) by DSM-IV criteria but subsequently re-categorized as BED by DSM-5 criteria, and consented to allow their data to be used for research purposes. Exclusion criteria was if the patient had been previously diagnosed with another eating disorder, such as AN or BN at the center.

### Measures

The *Eating Disorder Examination Questionnaire* (EDEQ; Fairburn and Beglin, 1994) assesses cognitions and behaviors associated with EDs and is a 36-item questionnaire adaptation of the Eating Disorder Examination structured interview (Fairburn and Cooper, 1993). The item assessing number of days of binge eating in the past month was used in the current study to characterize average binge eating symptoms in the BED sample. In our sample, 13% of patients were missing data on this variable.

The *Eating Disorder Inventory-II* (EDI-II; Garner, 1991). The EDI-II is a 91-item questionnaire frequently used to measure ED psychopathology. The questionnaire is divided into 12 scales assessing different aspects of disordered eating. Higher scores on the scales represent higher levels of eating disordered attitudes and behaviors. Most EDI-II scales had acceptable internal consistency and re-test stability as reported in a previous study on BED (Tasca et al., 2003). Five EDI-II scales were used in this study. The Interpersonal Distrust and Social Insecurity scales indicate problems in social relationships. The Ineffectiveness scale indicates a low level of self-esteem and a propensity for self-blame. The Asceticism scale measures a tendency to deprive oneself of pleasure. Finally, the Impulse Regulation scale measures a tendency to act out one’s impulses. In our sample, approximately 4% of patients were missing data on EDI-II subscales.

The EDI-II was used to compare our BED sample to a sample of mixed ED patients comprised of patients with AN and BN (*N* = 889) published in Garner (1991). The mixed ED comparison sample was composed of 129 individuals with AN – Restricting type, 103 individuals with AN – Binge-eating/Purging type, and 657 individuals with BN. Gender breakdown of this comparison sample was not available. The average age for these clinical samples was approximately 23. The EDI-II was also used to compare our BED sample to a sample of female non-patients (*N* = 205) also published in Garner (1991). The female non-patient comparison sample was made up of females that were recruited from the Michigan State University, with a corresponding average age of 19.9 (*SD* = 3.0).

The *Experiences in Close Relationships Scale* (ECR; Brennan et al., 1998) is a 36-item self-report measure with two scales. The Attachment Anxiety scale assesses concern with interpersonal rejection, and preoccupation with relationships, in which higher scores indicate greater attachment anxiety. The Attachment Avoidance scale measures fear of intimacy, and discomfort with closeness or dependency in relationships, in which higher scores indicate greater attachment avoidance. Cronbach’s alpha for the avoidance subscale (α = 0.90) and for the attachment anxiety scale (α = 0.85) were acceptable in our sample using a cutoff of 0.70 (Connelly, 2011). Since this questionnaire was administered only between the years 2006 and 2015 in the center, there were only 152 patients with data (with 13% missing data for patients seen for consultation between those years).

A normative sample for comparison on the ECR is the non-patient sample of college-aged females published in Shaver et al. (2005). Means and standard deviations for female ED comparison samples for AN, BN, and mixed ED are found in Tasca et al. (2006) and sample recruitment information is available in Tasca et al. (2009). The mixed ED sample was composed of 98 patients with EDNOS, 74 with AN, and 138 with BN seen at the center between 2006 and 2008. The ED mixed-sample comparison groups had a mean age of 26.31 (*SD* = 8.76), mean body mass index (BMI; kg/m^2^) of 21.88 (*SD* = 6.20), and mean chronicity of ED symptoms of 7.46 years (*SD* = 7.6). Most of the sample was Caucasian, and had completed university.

The *Personality Assessment Inventory* (PAI; Morey, 1991) is a 344-item self-report questionnaire measuring both personality and psychopathology. It is made up of four validity scales, 11 clinical scales, and 5 treatment consideration scales. While the PAI is not used to diagnose individuals with a mental disorder, it does indicate an elevation in behaviors and cognitions relative to a standardized census-based normative non-clinical sample of 1000 United States adults. Scores for the normative sample are transformed to *T*-scores with *M* = 50 and *SD* = 10. This measure was validated in ED samples with scales showing acceptable internal consistency (Tasca et al., 2002). Six PAI scales were used in this study. The Nonsupport scale indicates the degree to which one perceives social supports as lacking or unavailable. The Drug Problem and the Alcohol Problem scales evaluate difficulty with use of substances. Elevation on the Paranoia scale is consistent with externalizing one’s problems by blaming or focusing on others. The Treatment Rejection scale measures one’s resistance to treatment. The Depression and Anxiety scales indicate subjective distress related to mood and anxiety.

Out of the entire sample of 424, 13 people had missing PAI questionnaires. Patients were further excluded based on scores on the four validity scales that indicate an invalid profile: inconsistency (ICN), infrequency (INF), negative impression management (NIM), and positive impression management (PIM). 16 people had high ICN scores that excluded them from the analysis. An additional six people were excluded because of elevated INF scores. Furthermore, 17 participants were excluded for extreme NIM and an additional 2 people were excluded in analyses because of exceptionally high PIM scores. The total number of people excluded because of scores on invalidity scales was 41 (10%), leaving a total of 370 useable test scores. Valid records represent about 90% of the completed administered tests which is a rate higher than is reported in previous studies (Schinka, 1995; Tasca et al., 2002).

The ED normative samples with PAI data that were used for comparison in this study were previously published (Tasca et al., 2002). They included female adult patients with valid PAI profiles and the following diagnoses: AN-R (*N* = 41), AN-B (*N* = 44), and BN (*N* = 84) seen in consultation at the same centre as the BED sample. Participants in the ED comparison groups were predominantly Caucasian with some college or university. The mean age for the AN-R comparison group was 23.2 (*SD* = 7.1), and 28.1 (*SD* = 8.8) for the AN-B group, and 29.6 (*SD* = 8.8) for the BN group.

#### Assessment of Patient Domains

Since this is a chart review study, we were limited in the measures available to us to define the six patient domains. However, through the research center’s clinical database we had access to demographics, consultation reports, the EDI-II, the ECR, and the PAI. Data bias was controlled for by choosing the measures used in each domain *a priori* to conducing the data analysis.

The *functional impairment domain* was assessed with items from the demographic questionnaire on family income (10.8% missing data), employment status (9.2% missing data), marital status (6.6% missing data), and highest level of education (less than 5% missing data). Comparison statistics were obtained from publicly available Canadian census data (Statistics Canada, 2017a,b,c,d) in which we made every attempt to match for sex and age when possible.

The *social support and interpersonal functioning domain* was assessed with five scales. First, the EDI-II Interpersonal Distrust scale, which assesses a person’s feelings of alienation and reluctance to form close relationships and reluctance to express thoughts or feelings to others (Garner, 1991). Second, the EDI-II Social Insecurity scale, which assesses the belief that one’s social relationships are tense, insecure, disappointing, unrewarding, and poor quality (Garner, 1991). Third, the ECR Attachment Anxiety scale, which assesses preoccupation with relationships (Brennan et al., 1998). Fourth, the Attachment Avoidance scale, which assesses a tendency to dismiss relationships (Brennan et al., 1998). And fifth, the PAI Nonsupport scale, which assesses a lack of social supports (Morey, 1991).

The *problem complexity and chronicity domain* was assessed with the PAI Drug Problem and Alcohol Problem scales, as substance use disorders are frequently comorbid with BED (Hudson et al., 2007; Grilo et al., 2009; Gerlach et al., 2016). Although mood, anxiety, and personality disorders also commonly occur in BED, the mood and anxiety scales will be reported on within the subjective distress domain for this study so as not to duplicate analyses. Age of onset of binge eating was extracted from the consultation reports in order to assess chronicity of BED (missing for 7.8% of the patients).

The *coping style domain* was assessed with several scales. To assess internalizing coping we used the EDI-II Ineffectiveness scale, which measures aspects of internalizing such as feelings of inadequacy, worthlessness, and lack of control over one’s life and the EDI-II Asceticism scale, which measures aspects of internalizing such as pursuit of virtue via self-discipline, self-denial, restraint, self-sacrifice, and self-control (Garner, 1991). To assess externalizing coping we used the EDI-II Impulse Regulation scale, which measures aspects of externalizing coping such as recklessness, hostility, and relational destructiveness, and the PAI Paranoia scale which in part assesses a tendency to blame others.

The patient *resistance domain* was assessed with the PAI Treatment Rejection scale, which assesses one’s openness to accept treatment and recommendations. Finally, the *subjective distress domain* was assessed with the PAI Depression scale and PAI Anxiety scale, which are common indicators of subjective distress.

### Procedures

Two doctoral-level clinicians independently diagnosed each participant in separate semi-structured clinical interviews based on DSM criteria and on the Eating Disorder Examination diagnostic items (EDE; Fairburn and Cooper, 1993). The clinicians reached consensus about diagnoses and reported them in a consultation report. Previous research at our center indicated that this method of coming to an ED diagnosis results in very high inter-rater agreement with independent judges (Illing et al., 2011). All of those who met criteria for BED under DSM-IV/DSM-IV-TR (i.e., assessed between 1997 and 2013; *N* = 365) by definition also met criteria for BED in DSM-5. Charts of those diagnosed with EDNOS under DSM-IV were reviewed to assess if the patient met criteria for BED under DSM-5. Fifty-nine cases of these cases were re-assigned a diagnosis of BED under DSM-5 criteria. Participants who met DSM-5 criteria for BED (*N* = 424) completed questionnaires prior to receiving any treatment. Body Mass Index (BMI) was assessed during the consultation appointment by a member of the eating disorder program staff using a calibrated medical scale to measure weight and height. The Ottawa Health Sciences Network Research Ethics Board approved this study and all patients provided written, informed consent for their data to be used for research.

### Plan of Analysis

Most of the analyzes for this study involved comparison of means between the BED sample and previously published normative or ED samples using independent samples *t*-tests. In the case of comparing rates or frequencies to population-level data, we used Chi-Square tests. In order to correct for inflation of Type I error, a Holm–Bonferroni correction (Holm, 1979) was applied to multiple comparisons within each of the six patient domains of interest. We also corrected inflated Type I error due to multiple Spearman correlations between BED severity and variables associated with each domain by using the Holm–Bonferroni correction. Wherever possible, effect sizes are reported for independent-sample comparisons using Cohen’s *d* in which a *d* > 0.20 is a small effect, *d* > 0.49 is a medium effect, and *d* > 0.79 is a large effect (Cohen, 1988). Analyses were run using SPSS statistical software, version 21.

Scales with more than 5% missing data were assessed for missingness patterns using BMI as the index variable. BMI is a proxy for disorder severity and was available for all 424 participants with BED. In all cases, data were found to be missing at random.

## Results

### Characteristics of the BED Sample

Demographic variables are reported in Table 1, which also contains information on BMI and disorder status. The average patient was morbidly obese and the vast majority had current active symptoms. Using the ordinal severity scale from the EDEQ for number of binge days in the past 28 days in which 0 = 0 days binged, 1 = 1–5 days binged, 2 = 6–12 days binged, 3 = 13–15 days binged, 4 = 16–22 days binged, 5 = 23–27 days binged, 6 = binged every day, we found that the mean severity rating was 3.18 *SD* = 1.88 (*N* = 369). This indicated that on average patients with BED binged between 13 and 15 days per month. The number of days binged can be used as a metric for disorder severity (American Psychiatric Association, 2013). Days binged, treated as an ordinal variable, was correlated with each assessment variable within each domain and results are also presented below.

### Hypothesis 1: Functional Impairment Domain

Results mostly supported hypothesis 1. Individuals with BED showed a higher level of functional impairment compared to the general population. The BED sample attained a lower level of education compared to the Canadian national rates of educational attainment of those aged 25–64 in Canada (64.1%; Statistics Canada, 2017b), *X*^2^(1, *N* = 344) = 10.728, *p* = 0.001. The median response category for annual family income for the BED sample was lower than the median annual family income in the city in which most of the participants resided ($74,500; Statistics Canada, 2017d). However, the number of employed persons among the BED sample was not significantly lower than the July 2017 municipal employment rate for adults aged 15 and older (64.4%; Statistics Canada, 2017b), *X*^2^(1, *N* = 384) = 3.760, *p* > 0.05. Using the Chi-Square test on counts of patients in each of the marital status categories (Table 1) to compare to the municipal breakdown (Statistics Canada, 2017a), we found that frequencies for our sample with BED were significantly different from the available municipal statistics, *X*^2^(5, *N* = 396) = 35.274, *p* < 0.001. Less than the expected proportion of patients with BED were married (38.9%) compared to the population (47.4%), and almost double the expected proportion of those with BED were currently divorced (11.1%) compared to the population (6%). In our sample, severity of BED, as measured by days binged, was not significantly correlated to any of the functional impairment metrics.

According to a meta-analysis (Beutler et al., 2002), those with more functional impairment have poorer treatment outcomes, and may need more intense treatments such as intensive psychotherapy. Given that those with BED were found to be more functionally impaired compared to the general population on three different measures of functional impairment (educational attainment, family income, marital status), treatment-seeking patients with BED may require more intensive treatment.

### Hypothesis 2: Social Support and Interpersonal Functioning Domain

The findings supported hypothesis 2 related to social support and interpersonal functioning. Women with BED compared to their non-patient female counterparts had higher levels of Interpersonal Distrust and Social Insecurity (Table 2). Also, the BED sample had significantly lower levels of Interpersonal Distrust and Social Insecurity than the mixed ED sample published in the EDI-II manual (Table 2; Garner, 1991). As indicated in Table 3, those with BED reported higher levels of Attachment Anxiety and Attachment Avoidance than a non-clinical sample, but similar levels compared to other ED diagnostic groups. Finally, those with BED reported significantly higher scores on the Nonsupport scale of the PAI compared to a United States normative sample (Table 4), but there was no significant difference between those with BED and clinical ED samples once the Holm–Bonferroni correction was applied. A meta-analysis indicated that low levels of social support are associated with less favorable treatment outcomes and higher rates of relapse (Beutler et al., 2002). BED severity, measured as days binged per month, was significantly correlated to one the subscales in this domain: the Social Insecurity subscale (*r* = 0.159, *p* = 0.003). Hence, the presence of significant problems in the social support and interpersonal functioning domain suggests that treatment-seeking patients with BED may respond well to interpersonally oriented or social-skills-oriented treatments.

**TABLE 2:** Comparison of BED sample to non-clinical and eating disordered samples on EDI-II scales.

	**BED**			**Comparison sample**				
				
**EDI-II scale**	***N***	***M***	***SD***	***N***	***M***	***SD***	***d***	***p***
	**Female BED**			**Female non-patient^a^**				
				
Interpersonal distrust	365	4.2	4.0	205	2.0	3.1	0.61	<0.001
Social insecurity	363	6.6	4.6	205	3.3	3.3	0.82	<0.001
Ineffectiveness	363	9.5	7.1	205	2.3	3.6	1.28	<0.001
Asceticism	363	6.9	3.6	205	3.4	2.2	1.17	<0.001
Impulse regulation	361	3.9	4.6	205	2.3	3.6	0.39	<0.001

	**BED**			**Mixed ED^a^**				
				
Interpersonal distrust	411	4.3	4.0	889	5.8	4.7	0.34	<0.001
Social insecurity	409	6.7	4.6	107	8.6	4.9	0.40	<0.001
Ineffectiveness	409	9.4	7.1	889	11.3	7.8	0.25	<0.001
Asceticism	409	6.8	3.6	107	8.3	4.7	0.36	<0.001
Impulse regulation	407	4.0	4.5	107	6.0	5.3	0.41	<0.001

*EDI-II, eating disorders inventory-II; ED, eating disorder. All independent samples *t*-tests corrected with a Holm–Bonferroni correction. *d* = Cohen’s *d* effect size estimate. ^*a*^The female non-patient and mixed ED comparison sample data come from Garner (1991).*

**TABLE 3:** Comparison of BED sample to non-clinical and eating disordered samples on attachment scales to assess social support and interpersonal functioning.

	**Comparison samples**				
			
**ECR scales**	***N***	***M***	***SD***	***d***	***p***
**BED**					
Attachment anxiety	152	4.28	1.37		
Attachment avoidance	152	3.46	1.42		
**Female non-patient^a^**					
Attachment anxiety	72	3.55	1.19	0.57	**<0.001**
Attachment avoidance	72	2.03	0.72	1.27	**<0.001**
**AN^b^**					
Attachment anxiety	74	4.19	1.25	0.07	0.634
Attachment avoidance	74	3.55	1.38	0.06	0.652
**BN^b^**					
Attachment anxiety	138	4.36	1.29	0.06	0.610
Attachment avoidance	138	3.93	1.28	0.35	**0.003**
**Mixed ED^b^**					
Attachment anxiety	310	4.26	1.24	0.02	0.875
Attachment avoidance	310	3.76	1.25	0.22	0.021

*ECR, experiences in close relationships questionnaire; AN, anorexia nervosa; BN, bulimia nervosa; ED, eating disorder. All independent samples *t*-tests corrected with a Holm–Bonferroni correction. Significant differences after correction are in boldface. *d* = Cohen’s *d* effect size estimate. ^*a*^The female non-patient sample data comes from Shaver et al. (2005). ^*b*^The AN, BN, and mixed ED samples of women are described in Tasca et al. (2009) and data are from Tasca et al. (2011).*

**TABLE 4:** *Personality Assessment Inventory* scale scores for BED sample compared to normative and other eating disorder samples.

	**BED *N* = 370**	**Normative^a^*N* = 1000**				**AN-R^b^*N* = *41***				**AN-B^b^*N* = 44**				**BN^b^*N* = 84**		
								
**PAI subscale**	***M(SD)***	***t***	***p***	***d***	***M(SD)***	***t***	***p***	***d***	***M(SD)***	***t***	***p***	***d***	***M(SD)***	***t***	***p***	***d***
Nonsupport	56.6 (11.8)	10.3	**<0.001**	0.60	55.4(16.0)	0.59	0.553	0.09	60.9(15.5)	2.20	0.028	0.10	58.8(14.2)	1.48	0.139	0.17
Drug problem	51.4(10.9)	2.17	0.030	0.13	52.6(13.7)	0.68	0.498	0.09	51.9(10.0)	0.32	0.750	0.05	52.9(12.7)	1.14	0.255	0.13
Alcohol problem	48.4(9.6)	2.73	**0.006**	0.16	46.3(6.20)	1.34	0.180	0.26	48.4(8.90)	0.03	0.979	0.00	52.2(13.0)	3.08	**0.002**	0.33
Paranoia	56.3(11.7)	5.37	**<0.001**	0.33	53.2(12.8)	0.03	0.029	0.32	56.8(11.9)	2.19	0.029	0.32	55.9(10.5)	2.18	0.030	0.25
Treatment rejection	34.7(8.54)	26.2	**<0.001**	1.65	32.5(7.40)	1.57	0.117	0.28	32.7(7.10)	1.48	0.140	0.25	32.5(7.4)	2.16	0.031	0.28
Depression	67.7(13.4)	26.3	**<0.001**	1.50	72.4(18.2)	2.07	**0.039**	0.29	77.9(17.7)	4.62	**<0.001**	0.65	73.3(14.9)	3.41	**<0.001**	0.40
Anxiety	62.5(12.5)	19.2	**<0.001**	1.10	69.6(17.5)	3.27	**0.001**	0.47	74.1(13.8)	5.72	**<0.001**	0.88	71.3(12.0)	5.83	**<0.001**	0.72

*PAI, Personality Assessment Inventory; AN-R, anorexia nervosa, restricting; AN-B, anorexia nervosa, binge-purge; BN, bulimia nervosa. All independent samples *t*-tests corrected with a Holm–Bonferroni correction. Significant differences after correction are in boldface. *d* = Cohen’s *d* effect size estimate. ^*a*^The PAI United States normative sample data comes from the PAI manual (Morey, 1991) where all normative means are standardized to *M* = 50.0 and *SD* = 10.0. ^*b*^The AN-R, AN-B, and BN samples are female-only and data come from Tasca et al. (2002).*

### Hypothesis 3: Problem Complexity/Chronicity Domain

The findings regarding complexity and chronicity provided mixed support for hypothesis 3. Patients with BED had more complex mental health problems than the general population and somewhat less complex than those with AN-B and BN. Out of the 414 patients with BED for whom medical charts contained information on psychiatric comorbidities, current mood and anxiety disorders were relatively common (46.6 and 17.1%, respectively) and statistically significantly higher than the national 1-year prevalence rate in Canada for mood disorders at 8.3% (Health Canada, 2002), *X*^2^(1, *N* = 414) = 838.900, *p* < 0.001, and for anxiety disorders at 12.2% (Health Canada, 2002), *X*^2^(1, *N* = 414) = 10.397, *p* = 0.001. Notably, 13.8% of those with BED had more than one current comorbid mental health condition. The rate of antidepressant medication-use was statistically significantly higher in our BED sample (54.8%) compared to a 12-month prevalence rate of 5.8% calculated from a survey from 2002 on Canadian adults (Beck et al., 2005), *X*^2^(1, *N* = 414) = 1581.417, *p* < 0.001. We also examined problem complexity in the BED sample by looking at elevations of PAI scales related to substance use (Table 4). The BED sample did not report significantly higher problems with drugs compared to United States population norms but did score significantly lower on alcohol problems than the United States population norm (Morey, 1991) and compared to those with BN (Tasca et al., 2002). The median chronicity of binge-eating symptoms was 11–20 years, indicating that the vast majority of patients seeking treatment for BED reported chronic symptoms. More than 95% of the BED sample reported BED symptoms that were chronic (>1 year in duration). According to the meta-analysis by Beutler et al. (2002), the presence of multiple comorbidities in patients with psychological problems predicts poorer outcomes. In our sample, BED severity was not significantly correlated to any of the problem complexity metrics (i.e., chronicity, number of comorbidities, Alcohol Problems subscale, or Drug Problems subscale). The findings suggest that longer and more intense psychotherapy with the possible addition of medication could be used to address this risk among those with BED.

### Hypothesis 4: Coping Style Domain

Hypothesis 4 was supported indicating evidence for a propensity toward internalizing coping among those with BED. Compared to non-clinical groups, those with BED had higher scores on the EDI-II Ineffectiveness and Asceticism scales (Table 2), with large effects. Compared to the mixed ED sample, the BED sample had statistically significantly lower scores on both measures of internalizing. There was some evidence of elevated externalizing coping among those with BED, but the effects were small. With regard to the EDI-II Impulse Regulation scale and the PAI Paranoia scale, female patients with BED scored significantly higher than their female non-patient counterparts, with a small effect. Further, those with BED scored significantly lower than those with other EDs on Impulse Regulation, with a small effect (see Table 2). Severity of binge eating was significantly correlated to three subscale representing internalizing coping and externalizing coping: Ineffectiveness (*r* = 0.226, *p* < 0.001), Asceticism (*r* = 0.216, *p* < 0.001), and Impulse Regulation (*r* = 0.225, *p* < 0.001).

A meta-analysis by Beutler et al. (2018a) indicated that those with internalizing coping styles tend to respond better to insight focused treatments. Based on the finding that internalizing coping is the predominant method used by those with BED, it is likely that they may respond more favorably to interventions emphasizing gaining a better understanding of their disorder and its maintenance, rather than an exclusively symptom-focused approach.

### Hypothesis 5: Resistance Domain

The findings supported the hypothesis that patients with BED will have low levels of resistance to treatment. The BED sample scored statistically significantly lower on the Treatment Rejection scale of the PAI compared to the non-clinical normative sample, with a large effect, indicating an openness to therapy among those with BED. This score was not statistically significantly different from other treatment-seeking ED comparison groups (see Table 4). The findings suggest that those with BED who are seeking treatment are as likely to comply with treatment as others with an ED, and do not report significant problems with interpersonal resistance compared to the norm. BED severity was significantly and negatively correlated to the Treatment Rejection scale (*r* = −0.225, *p* < 0.001).

In line with the findings by Beutler et al.’s (2018b) meta-analysis on patient resistance, patients who experience low resistance to treatment are not likely to drop out of psychotherapy and may comply well with treatment recommendations and directives.

### Hypothesis 6: Subjective Distress

The results generally supported the hypothesis that, on average, those with BED experienced a moderately high level of distress and thus were likely motivated for treatment. Scores on PAI Depression and Anxiety scales were significantly higher than the United States normative population with medium to large effects. The BED sample had significantly lower scores compared to other treatment-seeking ED samples (see Table 4). Severity of BED was significantly correlated to both symptom distress subscales: Depression (*r* = 0.246, *p* < 0.001) and Anxiety (*r* = 0.196, *p* < 0.001).

A meta-analysis by Beutler et al. (2011) suggested that a medium level of subjective distress (i.e., not too high or too low) is compatible with making changes through treatment. Given that the BED sample demonstrated medium levels of distress, one can expect that the average patient with BED will be motivated for treatment and likely to follow treatment recommendations.

## Discussion

**FIGURE 1:**
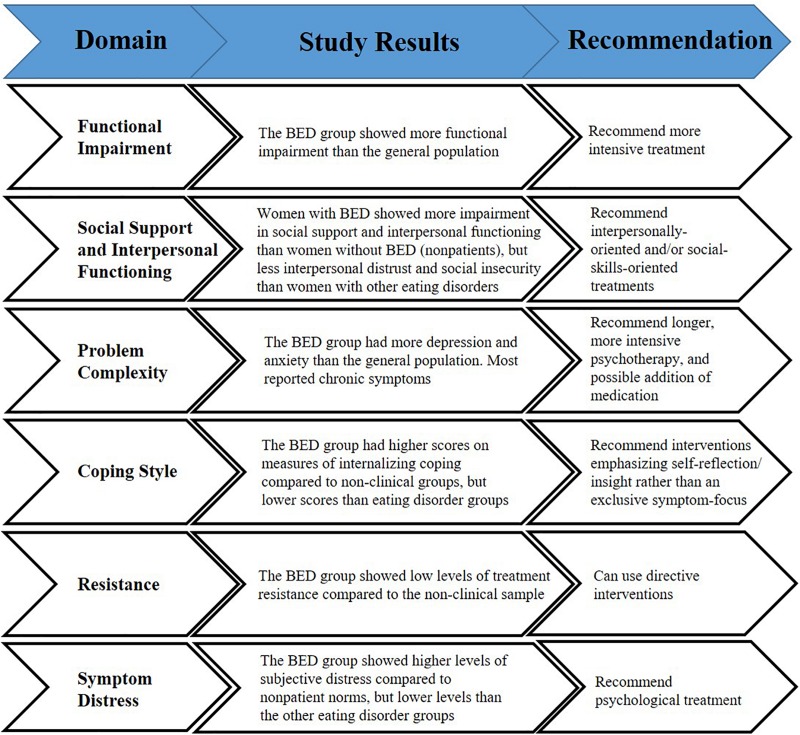
Summary of the clinical decision-making approach presented by domain.

This was an empirical study based on an evidence-based clinical decision-making approach to assessing treatment-seeking patients with BED (Harwood et al., 2011). Such an approach is consistent with a large body of research indicating that patient characteristics are by far the most predictive of psychological treatment outcomes (Norcross, 2011; Cuijpers et al., 2012). We compared a large sample of treatment-seeking patients with BED to population-based data, non-clinical normative data, and other treatment-seeking ED samples using validated psychometric tests when possible. Our intent was to inform general treatment recommendations for the average, treatment-seeking patient with BED in order to suggest future directions for clinical decision-making and idiographic case formulation that takes into account individual patient domains that are known to affect outcomes (Silberschatz, 2017). Binge-eating severity was associated with several of the patient domain indicators, suggesting that not only the diagnosis itself but severity of symptoms may be related to some of these patient factors. For a summary of the results and of the decision-making approach utilized, please see Figure 1.

### Functional Impairment Domain

In this study we used available objective indices to evaluate the severity of functional impairment found in those with BED. Our hypothesis that those with BED would demonstrate greater functional impairment than population averages was supported for the most part. Those with BED reported lower levels of educational attainment, lower family incomes, lower rates of marriage, and more instances of divorce. The findings are consistent with research indicating lower average family income among those with BED (Hay et al., 2017), however, no previous study of patients with BED reported population-based comparative data related to education and employment. These findings are generally consistent with patients who experience less favorable psychotherapy outcomes, and suggest the need for intensive psychological treatment and perhaps the use of adjunctive medical interventions (Beutler et al., 2002).

### Social Support and Interpersonal Functioning Domain

Low levels of social support are associated with less favorable treatment outcomes and higher rates of relapse in most clinical samples (Beutler et al., 2002). As predicted, those with BED had significantly higher levels of interpersonal distrust, social insecurity, attachment anxiety, attachment avoidance, and perceived non-support compared to non-clinical samples. Compared to a mixed treatment-seeking ED comparison sample, those with BED had slightly lower levels of interpersonal distrust and social insecurity, but similar levels on all other social and interpersonal problem indicators. These findings are consistent with previous reports of patients with BED (Ivanova et al., 2017; Maxwell, 2017). The interpersonal model of BED, which has been tested in empirical studies (e.g., Ivanova et al., 2017), suggests that interpersonal problems may lead to negative affect or affect dysregulation that then precipitate binge eating. Therapists treating those with BED may consider incorporating specific interventions that emphasize social skills and interpersonal functioning in order to reduce the impact of factors like negative affect that maintain binge eating.

### Problem Complexity/Comorbidity/Chronicity Domain

For most mental disorders, dual diagnoses and longer chronicity predict a poorer prognosis and the need for longer and more intensive treatment (Beutler et al., 2002). As expected, relative to the general population, those with BED had higher rates of mood and anxiety disorder diagnoses. The majority of those with BED were taking an antidepressant medication, and this percentage was also significantly greater than the population. This is consistent with previous population-based research that found that BED is associated with mood and anxiety disorders (Hudson et al., 2007; Kessler et al., 2013). Our sample of treatment-seeking individuals with BED showed similar levels of substance use behaviors and cognitions compared to the other ED groups. Previous research identified BN as the ED diagnosis associated with higher levels of substance abuse, and specifically alcohol abuse (Bulik et al., 2004; Ulfvebrand et al., 2015). Almost the entire sample with BED reported chronic symptoms of binge eating (>1 year in duration), which is understandable given that the patients were treatment-seeking. We advise clinicians of patients with BED to assess for comorbid conditions and, if necessary, to treat concurrently these conditions in order to increase the likelihood of positive outcomes for BED (Harwood et al., 2011).

### Coping Style Domain

The results supported the hypothesis that the BED sample would be more internalizing in their coping style than the non-patient norm. Binge eating symptoms and limiting awareness of emotions by internalizing may be ways that patients with BED cope with emotional distress and interpersonal conflict (Tasca and Balfour, 2014; Hill et al., 2015). However, the BED sample had similar or lower levels of internalizing compared to other treatment-seeking ED groups. This suggests that the internalizing coping of those with BED is a prominent mode of coping, but may not be as extreme as seen among those with other EDs. We made no hypotheses about the use of externalizing coping among those with BED but found evidence that the BED group engaged in more externalizing than the normative population, but these effects were small and so may not be clinically meaningful. Further, greater severity of binge eating had a small but significant association with higher internalizing and externalizing coping. Given these findings regarding coping style, one could argue that internalizing coping is most likely among average patients with BED. If that is the case, then patients may benefit from treatment that emphasizes insight into the causes or maintenance of binge eating symptoms (Beutler et al., 2018b), such as interpersonal distress and affect dysregulation (Ivanova et al., 2017).

### Social Compliance and Resistance Domain

In a meta-analysis, Beutler et al. (2018a) found that patients who were low in resistance were more likely to respond to more directive therapist behaviors, whereas those higher in resistance were more likely to respond to less directive therapists. The results of the current study supported the hypothesis that patients with BED would exhibit less treatment resistance than a non-clinical normative group, and less resistance than other ED treatment-seeking groups especially as symptom severity increases. Many treatment-seeking groups tend to score low on the PAI Treatment Rejection subscale (Harwood et al., 2011), but this is the first report that we know of to evaluate social compliance and resistance in those with BED. Based on the findings of this study, we expect that most patients with BED will show low resistance and respond well to direction in therapy. Therapists should assess for level of resistance in their patients with BED and adjust therapist level of directiveness accordingly.

### Subjective Distress Domain

Distress that is too low may lead to insufficient motivation to change, whereas distress that is too high may be too overwhelming for patients to make use of therapy (Harwood et al., 2011). Consistent with this notion of optimum level of distress, we found that those with BED had significantly higher levels of anxiety and depression compared to the norm, but slightly lower levels compared to those with AN-B and BN. Eating disorders are notoriously difficult to treat, with many patients experiencing ego-syntonic symptoms and low motivation to change that may result in poor treatment outcomes (Vitousek et al., 1998). The level of motivation of the average patient seeking treatment for BED in this study, as indexed by level of distress, appears to be within an optimal range and tends to increase with more severe binge eating symptoms. Nevertheless, therapists may need to assess motivation for treatment by using valid assessment approaches (Cassin et al., 2008) or by assessing overall distress. For patients with low motivation, the therapist may help to increase motivation using motivational enhancement techniques (Cassin et al., 2008), but for those with high levels of subjective distress therapists may need to intervene to reduce anxiety or depressive symptoms before engaging the patient in interventions for the binge eating.

### Limitations

While this study made use of a large sample size of patients with BED and a number of comparisons with normative and clinical ED samples, it is not without its limitations. Although our intention is to encourage clinicians to take an idiographic approach to assessing patient characteristics that impact treatment outcomes, our results necessarily speak to the average patient with BED. Findings presented in this study should not prevent clinicians from considering individual variability in assessing their patients on the six evidence-based domains for clinical decision-making and case formulation. Our conclusions are inferred from an established integrative model of clinical decision-making using patient factors that are based on meta-analytic evidence broadly applicable to mental health patients. Our sample is a diagnostically homogeneous sample for which the integrative model used may not necessarily apply. Due to the lack of available outcome data, we could not directly address whether these specific patient variables for clinical decision-making are useful in directing patients to treatments. More research is needed to examine which patient factors can best predict treatment outcomes for adults with BED so that they may receive the most appropriate care.

Other limitations relate to the measurement of constructs which are part of the integrative decision-making model and to the sample used were present. For example, unemployment rate, which was used to assess functional impairment, does not take into account those who are out of the labor force by choice (e.g., homemakers, students not looking for work, those who are retired, or others not seeking employment). Thus our findings may overestimate that number of patients who were unable to work because of functional impairment. Several of the measures used to represent concepts such as internalizing and externalizing coping, or resistance may only capture some aspects of the constructs and thus may not be fully representative. For example, a more specific self-report measure of coping, such as the Coping Orientations to Problems Experienced (Carver et al., 1989) may corroborate our findings. However, for most domains, we relied on several variables to assess the constructs and we used validated scales of those constructs to increase the validity of findings and reliability of measurements. Another limitation was that diagnostic information was based on semi-structured clinical interviews of patients based on the EDE questions (Fairburn and Cooper, 1993) to guide diagnoses. Nevertheless, the clinicians completing the interviews were experts working in a tertiary care center for EDs, and previous research in our center indicated that this method of coming to diagnoses is highly reliable (Illing et al., 2011). Finally, we were limited to published comparison groups which precluded matching of individuals in our sample on some demographic variables.

## Conclusion

Based on the results of these analyses, one can infer that the average treatment-seeking patient with BED: (1) is moderately functionally impaired relative to the normative population, thus possibly requiring intensive treatment; (2) has low social support and impaired interpersonal functioning, in a manner almost equivalent to those with more severe EDs, and so may need treatment targeting interpersonal and social issues; (3) presents with complex, comorbid, and chronic psychopathology, almost equivalent to those with other EDs, and so may need longer and concurrent treatment for the comorbid conditions; (4) may be more internalizing than externalizing in terms of coping style, and so may benefit from an approach that focuses on insight into causes and maintenance of the binge eating such as interpersonal problems and affect dysregulation; (5) is not resistant to treatment, and so might respond relatively well to more directive interventions; and (6) is optimally motivated to change suggesting less need for motivation enhancement or for reducing debilitating distress. For a summary, please see Figure 1. This study may help clinicians by providing general treatment recommendations for patients with BED (i.e., more intensive therapy; interventions targeting interpersonal and social issues; longer treatments that also focus on comorbid conditions; approaches that focus on insight; directive interventions; and not necessarily comprising motivational enhancement). These recommendations may be beneficial in the absence of assessment information specific to the individual patient with BED.

Despite the nomothetic nature of these findings, we encourage clinicians to recognize that there is variability among individuals who share mental health diagnoses, including those with BED. Treatments for BED are moderately effective and could be improved (Grenon et al., 2017). Psychotherapists can use information about patient functioning domains presented in this study during their assessments to develop ideographic case formulations (Silberschatz, 2017) of patients with BED. Such assessments and formulations using evidence-based patient factors will inform precision treatment of individual patients based on an integrative transtheoretical framework (Harwood et al., 2011) in order to improve outcomes for BED. While a focus on these explicit patient factors is recommended, clinicians should keep in mind that other variables such as childhood trauma may also help explain the variability in ED treatment outcomes (Mahon et al., 2001; Rodríguez et al., 2005), and are likely related to the six variables discussed in this study (van der Kolk et al., 2005).

We recommend that prior to offering treatment to those with BED an assessment of these evidence-based patient domains should take place to inform treatment planning. With a thorough assessment, therapists can optimally respond with the most effective interpersonal stances based on patient: level of functional impairment, social support, problem complexity/comorbidity/chronicity, coping style, social compliance and resistance, and subjective distress.

## Data Availability Statement

The raw data supporting the conclusions of this manuscript will be made available by the authors, without undue reservation, to any qualified researcher.

## Author Contributions

LC ran analyses and wrote the draft. GT provided conceptual framework and wrote and edited the manuscript. HB contributed to the design methodology and editing.

## Conflict of Interest

The authors declare that the research was conducted in the absence of any commercial or financial relationships that could be construed as a potential conflict of interest.
